# AI in the Loop: functionalizing fold performance disagreement to monitor automated medical image segmentation workflows

**DOI:** 10.3389/fradi.2023.1223294

**Published:** 2023-09-15

**Authors:** Harrison C. Gottlich, Panagiotis Korfiatis, Adriana V. Gregory, Timothy L. Kline

**Affiliations:** ^1^ Mayo Clinic Alix School of Medicine, Mayo Clinic, Rochester, MN, United States; ^2^ Department of Radiology, Mayo Clinic, Rochester, MN, United States; ^3^ Division of Nephrology and Hypertension, Mayo Clinic, Rochester, MN, United States

**Keywords:** deep learning, semantic segmentation, machine learning model performance, similarity metrics, epistemic uncertainty, convolutional neural networks, AI in the loop

## Abstract

**Introduction:**

Methods that automatically flag poor performing predictions are drastically needed to safely implement machine learning workflows into clinical practice as well as to identify difficult cases during model training.

**Methods:**

Disagreement between the fivefold cross-validation sub-models was quantified using dice scores between folds and summarized as a surrogate for model confidence. The summarized Interfold Dices were compared with thresholds informed by human interobserver values to determine whether final ensemble model performance should be manually reviewed.

**Results:**

The method on all tasks efficiently flagged poor segmented images without consulting a reference standard. Using the median Interfold Dice for comparison, substantial dice score improvements after excluding flagged images was noted for the in-domain CT (0.85 ± 0.20 to 0.91 ± 0.08, 8/50 images flagged) and MR (0.76 ± 0.27 to 0.85 ± 0.09, 8/50 images flagged). Most impressively, there were dramatic dice score improvements in the simulated out-of-distribution task where the model was trained on a radical nephrectomy dataset with different contrast phases predicting a partial nephrectomy all cortico-medullary phase dataset (0.67 ± 0.36 to 0.89 ± 0.10, 122/300 images flagged).

**Discussion:**

Comparing interfold sub-model disagreement against human interobserver values is an effective and efficient way to assess automated predictions when a reference standard is not available. This functionality provides a necessary safeguard to patient care important to safely implement automated medical image segmentation workflows.

## Introduction

Automated medical image segmentation techniques offer a wide range of benefits to healthcare delivery. Deep learning–based image segmentations have already shown application in many different areas of the body as well as image modalities (1–4). Segmentations can be used directly to measure organ volume or can be used for 3D modeling and printing, demonstrating to patients the anatomical basis of diseases as well as educating surgical trainees through high-fidelity simulations (5). Automated segmentations can also be one part of a workflow where segmentation predictions are further fed into additional models to classify pathology and to inform medical decision-making. A challenge of implementing such a workflow is the need for robust quality control of automated segmentations without the potentially infeasible burden of continuous human monitoring (6).

Given the potential value of medical image segmentation, the topic of model development has been intensely studied on internal datasets as well as in open-sourced challenges (7–9). Recently, the “no new U-Net model” (nnU-Net) framework has consistently produced winning submissions in a number of these open challenges (10). A contribution of nnU-Net is to automate many of the neural network design choices and training strategies, allowing researchers to focus on other barriers to clinical implementation. One significant barrier to clinical implementation of automated workflows is in cases where implemented models encounter data that are not represented well in the training set, otherwise referred to as “out-of-distribution” data. This is particularly an issue for models that belong in the U-Net family, since they will always make predictions on properly formatted input data without a measure of certainty in its output, potentially leading to catastrophic results in research or clinical decision-making unless there exists some oversight of automated processes. For example, a model trained on cross-sectional CT images would still yield a prediction if magnetic resonance (MR) data were accidentally used, despite not having seen MR image data before. This potential for mismatch between training and task data is a significant issue for clinical implementation of automated deep learning models that are unable to flag poor predictions (11, 12).

Epistemic uncertainty refers to the lack of knowledge of a model's own limitations due to limited training data (13–15). Researchers have proposed several approaches to address issues of out-of-distribution task data, including proactively identifying out-of-distribution data before a model is applied by using separate machine learning classification models and/or Monte Carlo methods (14–17). These methods are a form of “AI in the Loop” where separate automated model processes are inserted into workflows to automatically check predictions and flag where human intervention may be needed. A drawback of the previous works is that they add significant complexity to the clinical implementation of machine learning workflows by requiring a separate training and monitoring of these upstream models. Our team investigated how to achieve the same benefit of automatic clinical workflow monitoring using data available in segmentation models without needing for a separate model or reference segmentations.

In this paper, we propose an easily implemented framework to equip conventionally trained fivefold cross-validation models with the ability to monitor real-time predictions when reference standards are not available, similar to a clinical workflow. This AI in the Loop method is novel in being easily understandable and quickly computable while powerfully enabling a clinically implemented image segmentation workflow to have some form of discrimination in determining whether a prediction segmentation needs human review.

## Materials and methods

### Dataset

This multi-dataset retrospective study was approved by our institutional review board, was HIPAA compliant, and performed in accordance with the ethical standards contained in the 1964 Declaration of Helsinki. We used two internal data sets: (1) an MR abdomen dataset with labeled kidney and tumor and (2) a CT abdomen dataset with labeled kidney and tumor. In addition, the open-sourced KiTS21 dataset as described in the publication by Heller et al. (8) was used to demonstrate the out-of-distribution task data. Our internal datasets are described in detail with demographic data in Table 1.

**Table 1:** Internal dataset demographics.

	CT dataset	MR dataset
No. of subjects	350	350
Males	229	217
Females	121	133
Age*	63 ± 13 (19–88)	59 ± 14 (20–88)
Height (m^2^)*	1.72 ± 0.1 (1.43–2.04)	1.73 ± 0.1 (1.49–2.04)
Weight (kg)*	92.79 ± 25.02 (45–200)	90.17 ± 22.33 (46–190)
BMI (kg/m^2^)*	31.00 ± 7.34 (16–62)	30.13 ± 6.52 (17–57)

* Mean ± standard deviation.

### MR kidney tumor dataset

As part of a previously published study, 350 T2-weighted images with fat-saturation, coronal, abdominal/pelvis MR images were randomly sampled from a dataset of 501 patients, where 313 of the patients had undergone partial nephrectomy, and 188 of the patients had undergone radical nephrectomy between 1997 and 2014 (18). The segmentation of these images was performed in two parts. In the first step, the right and left kidneys were segmented using a previously trained U-Net-based algorithm (19, 20). Then, two urologic oncology fellows manually refined these automatic segmentations and segmented renal tumors. A total of 50 images were randomly selected from this dataset to comprise a test set that will be used to evaluate the model.

### CT kidney tumor dataset

Also a part of the previously referenced study, 350 images were randomly sampled from a collection of 1,233 non-contrast and different contrast phases of abdomen/pelvis CT images as part of the Mayo Clinic Nephrectomy Registry (18, 21). The images were from patients without metastatic lesions or positive lymph nodes at the time of radical nephrectomy between 2000–2017. Two urologic oncology fellows segmented the kidney and tumor masks using the segmentation software ITK-snap **RRID:SCR_002010** (version 2.2; University of Pennsylvania, Philadelphia, PA, USA) (22). Processing of these images included cropping around both kidneys and three slices above the slice of the upper pole of the kidney and three slices below the lower pole of the kidney. The scans were resampled to a coronal plane width of 256-pixel and a medial plane depth of 128-pixel, employing zero padding if images were smaller than this standard size. A total of 50 images were randomly selected from this dataset to comprise a test set that will be used to evaluate the model.

## Algorithm

### nnU-Net specifications

The nnU-Net preprocessing involves designating “T2” or “CT” default processing for each dataset. nnU-Net offers four different default model configurations: 2d, 3d_fullres, 3d_lowres, and 3d_cascade_fullres. The 3d_lowres and 3d_cascade_fullres configurations are designed to be run sequentially for image data that are too large for the 2d or 3d_fullres configurations to handle. We opted to use the 3d_fullres configuration since we found that it performs better than the 2d configuration based on the findings from our previous work (18).

Following nnU-Net's public GitHub **RRID:SCR_00263** (23), a standard fivefold cross-validation process was utilized using the 3d_fullres configuration. In this process, the final predictions are derived by averaging the five sub-model outputs, which are the voxel-wise softmax probabilities, into one ensemble prediction (10). In addition, each sub-model prediction was evaluated to assess fold disagreement.

### Self-informed models

The main goal of this study is to utilize the information encoded in models generated during the fivefold cross-validation process to investigate whether information extracted during the inference stage can inform the end user of the segmentation quality of the final ensemble model.

During training, a fivefold cross-validation approach was utilized, generating five sub-models. In this paper, we define a sub-model as a fully trained model that has a unique training and validation set split. In nnU-Net's implementation of the fivefold cross-validation process, the predictions from the five sub-models on a test image are ensembled by averaging the voxel-wise softmax probabilities, in which the averaged voxel value is rounded to the nearest prediction value for predicting the final ensemble. In our method, we calculated Dice scores between each sub-model prediction, i.e., Dice between sub-model 1 and sub-model 2, between sub-model 1 and sub-model 3, and so on. Dice score is a commonly used metric to compare 3D image segmentations, where a score of 1 indicates complete overlap between the two segmentations and a score of 0 indicates two segmentations with no overlap (24). This process produced 10 Dice metrics referred to as “Interfold Dices” that were summarized by employing different first order summary statistics to compare against published human–human interobserver thresholds described below. As part of our investigations of metrics that can be used to flag cases that the ensemble model's prediction might be suboptimal on, the following first order statistics were evaluated: mean, median, minimum, and maximum of the Dice index.

We compared the summarized Interfold Dices with previously published human interobserver thresholds to evaluate whether disagreement between the folds was within the expected variance of a task or indicative of a lack of representative training data. We used a threshold of 0.825 for the MR kidney tumor task based on the work of Muller et al. (25). In this publication, the researchers reported human interobserver values of 0.87 and 0.78 in a dataset of a series of MR imaging from 17 patients with Wilms tumor before and after undergoing chemotherapy, respectively. We averaged these values arriving at the 0.825 threshold employed in our work. For the CT kidney tumor task, we used two studies to inform our threshold. In a study analyzing the effect of contrast phase timing on texture analysis to predict renal mass histology from CT scans, Nguyen et al. (26) reported an interobserver variability of 0.91–0.93 in a dataset of 165 patients. In a study including renal, liver, and lung pathologies (including the 300 sample KiTS19 dataset), Haarburger et al. (27) reported a median interobserver threshold of 0.87. We averaged these values deriving the 0.90 threshold employed in our study. In a sensitivity analysis for the KiTS21 task, we also investigated how the process of changing the threshold would affect the results of the method. In general, it was found out that a higher threshold will flag more images, both true and false positives, and can be tuned to a specific task in the model training phase.

To validate our method, we compared the summarized Interfold Dice with the final test ensemble Dice score to investigate whether an association existed. We first created scatter plots, where the y-axis was a given summary metric of the Interfold Dice and the x-axis was the Dice score of the final ensemble model. Intuitively, we also used confusion matrices to display the results, where true positives were flagged images based on the summary of Interfold Dice of the ensemble model with poor performance, true negatives were non-flagged segmentations of the ensemble model with good performance, false positives were flagged images of the ensemble model with good performance, and false negatives were non-flagged images of the ensemble model with poor performance. Of these categories, false negatives were considered the worst failure since they represented non-flagged poor segmentations that might not be reviewed before being utilized in a clinical workflow. False positives were undesirable but not evidently worrisome in small quantities since they would represent flagged images that had good performance and could be quickly reviewed. We also calculated how the overall test Dice set score would change if the flagged segmentations were removed.

### Simulating out-of-distribution task data

To test the generalizability of our framework in identifying “out-of-distribution” data, we used our internally trained model to predict segmentations on the open-sourced KiTS21 dataset (17), knowing that key differences existed between the datasets. The CT images in the KiTS21 dataset were all acquired from contrast-enhanced CT scans during the corticomedullary contrast phase, and these images contained generally smaller tumors including those from partial nephrectomies. In contrast, our internal dataset contained a mix of different contrast phases and had larger tumors being solely from a radical nephrectomy database. The difference in voxel dimensions and distribution of tumor size between the KiTS21 dataset and our internal dataset can be found in Table 2.

**Table 2:** Dataset voxel and volume characteristics.

	MR kidney tumor	CT kidney tumor	KiTS21
In-plane voxel width × height (mm)			
Mean	1.34 × 1.34	1.03 × 1.03	0.79 × 0.79
Median	1.56 × 1.56	1.01 × 1.01	0.78 × 0.78
Range	0.59–1.95 × 0.59–1.95	0.49–1.85 × 0.49–1.85	0.44–1.04 × 0.44–1.04
Slice thickness (mm)			
Mean ± SD	6.26 ± 1.65	4.03 ± 1.38	3.18 × 1.75
Median	6.00	5.00	3.00
Range	2.00–15.00	0.65–8.00	0.50–5.00
Number of slices			
Mean ± SD	32.27 ± 11.64	44.50 ± 23.91	314.67 ± 37.83
Median	30.00	37.00	320.00
Range	6.00–116.00	20.00–211.00	257.00–478.00
Volume of labels (ml)			
Tumor minimum volume	0.01	0.513	1.86
Tumor 25th percentile volume	29.61	55.95	18.71
Tumor mean ± SD	541.33 ± 1,244.35	428.61 ± 654.27	253.59 ± 476.38
Tumor median volume	97.33	212.71	66.77
Tumor 75th percentile volume	454.70	497.67	219.46
Tumor maximum volume	11,946.84	7,742.26	2,894.02

## Results

### MR kidney tumor results

**Figure 1:**
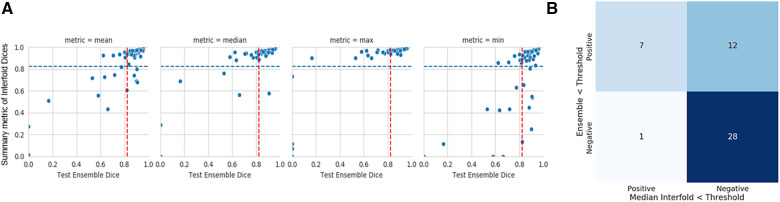
MR kidney tumor characteristics of flagged and non-flagged images. (**A**) Mean, median, maximum, and minimum Interfold Dice score plots. The blue dashed line indicates the interfold cutoff at the human threshold interobserver value (IO) (images below the line are flagged). The red dashed line indicates the ensemble IO performance (images to left of the line have low performance). (**B**) Confusion matrix for median Interfold Dice. True positive (upper left) is defined as when flagged images (summary Interfold Dice < threshold) performed poorly (test ensemble < threshold). True negative (lower right) is defined as when non-flagged images (summary Interfold Dice > threshold) performed well (test ensemble > threshold). False positives (upper right) defined as when flagged images (summary Interfold Dice < threshold) performed well (test ensemble > threshold). False negatives (lower left) defined as when non-flagged images (summary Interfold Dice > threshold) performed poorly (test ensemble < threshold).

In our study, the performance of the ensemble model on the holdout test set without flagging for MR kidney tumor was 0.76 ± 0.27. As described in the description of our method above, we used a flagging threshold of 0.825, where images with summarized Interfold Dices below this value were flagged. The full results of the impact of flagging with different summary metrics can be found below in Table 3 and Figure 1. All unflagged cohorts mean ensemble Dice values were above the human interobserver value with small standard deviations.

**Table 3:** Different summary metrics of interfold Dice flagged cohort—MR kidney tumor.

Summary metrics	Number of flagged images	Flagged images mean ± standard deviation	Non-flagged images mean ± standard deviation
Mean	17	0.53 ± 0.37	0.87 ± 0.07
Median	8	0.28 ± 0.36	0.85 ± 0.09
Max	4	0 ± 0	0.82 ± 0.15
Min	20	0.57 ± 0.36	0.87 ± 0.08

### CT kidney tumor results

**Figure 2:**
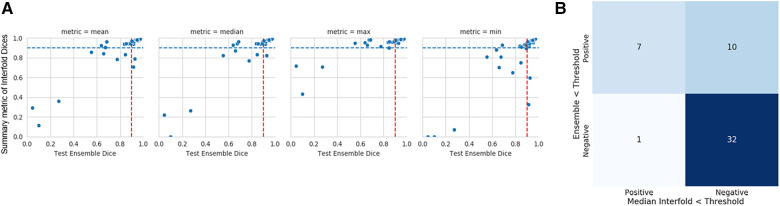
CT kidney tumor characteristics of flagged and non-flagged images. (**A**) Mean, median, maximum, and minimum Interfold Dice score plots. The blue dashed line indicates the interfold cutoff at IO (images below the line are flagged). The red dashed line indicates the ensemble IO performance (images to left of the line have low performance). (**B**) Confusion matrix for median Interfold Dice. True positive (upper left) is defined as when flagged images (summary Interfold Dice < threshold) performed poorly (test ensemble < threshold). True negative (lower right) is defined as when non-flagged images (summary Interfold Dice > threshold) performed well (test ensemble > threshold). False positives (upper right) defined as when flagged images (summary Interfold Dice < threshold) performed well (test ensemble > threshold). False negatives (lower left) defined as when non-flagged images (summary Interfold Dice > threshold) performed poorly (test ensemble < threshold).

The mean ensemble ± standard deviation Dice model performance for CT kidney tumor on the holdout test set was 0.85 ± 0.20. As described in the description of our method above, we used a threshold of 0.90, where images with summarized Interfold Dices below this value were flagged. The full results of the impact of flagging with different summary metrics can be found in Table 4 and Figure 2. Almost all the mean ensemble Dice values of the non-flagged cohorts were above the human interobserver value with small standard deviation values.

**Table 4:** Different summary metrics of interfold Dice flagged cohort—MR kidney tumor.

Summary metrics	Number of flagged images	Flagged images mean ± standard deviation	Non-flagged images mean ± standard deviation
Mean	9	0.57 ± 0.35	0.91 ± 0.08
Median	8	0.52 ± 0.34	0.91 ± 0.08
Max	4	0.32 ± 0.37	0.89 ± 0.10
Min	12	0.61 ± 0.31	0.92 ± 0.05

### Predictions on KiTS21 results

**Figure 3:**
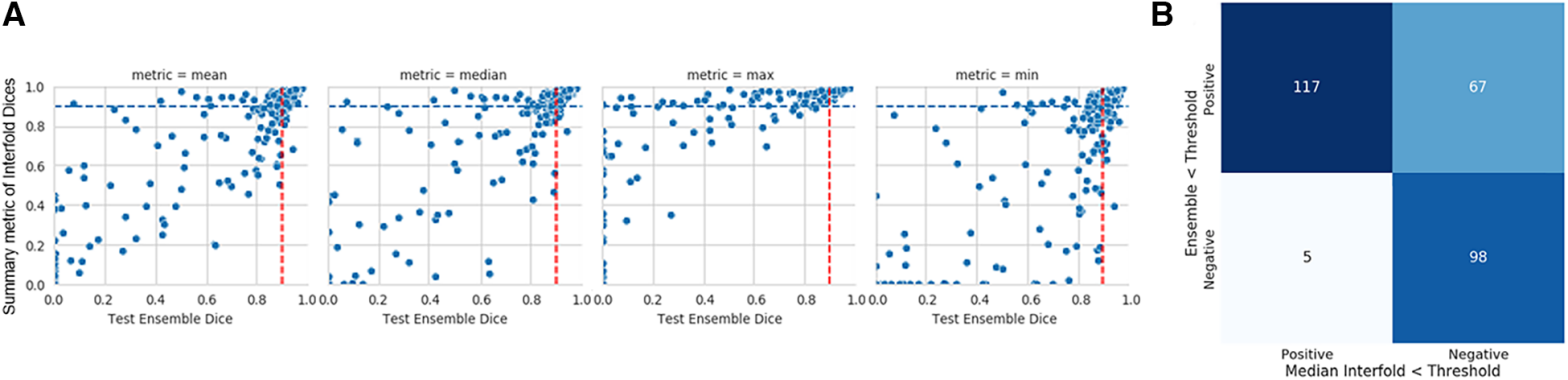
Internal CT kidney tumor model on KiTS21 data. (**A**) Mean, median, maximum, and minimum Interfold Dice score plots. The blue dashed line indicates the interfold cutoff at IO (images below the line are flagged). The red dashed line indicates the ensemble IO performance (images to left of the line have low performance). (**B**) Confusion matrix for median Interfold Dice. True positive (upper left) is defined as when flagged images (summary Interfold Dice < threshold) performed poorly (test ensemble < threshold). True negative (lower right) is defined as when non-flagged images (summary Interfold Dice > threshold) performed well (test ensemble > threshold). False positives (upper right) defined as when flagged images (summary Interfold Dice < threshold) performed well (test ensemble > threshold). False negatives (lower left) defined as when non-flagged images (summary Interfold Dice > threshold) performed poorly (test ensemble < threshold).

**Figure 4:**
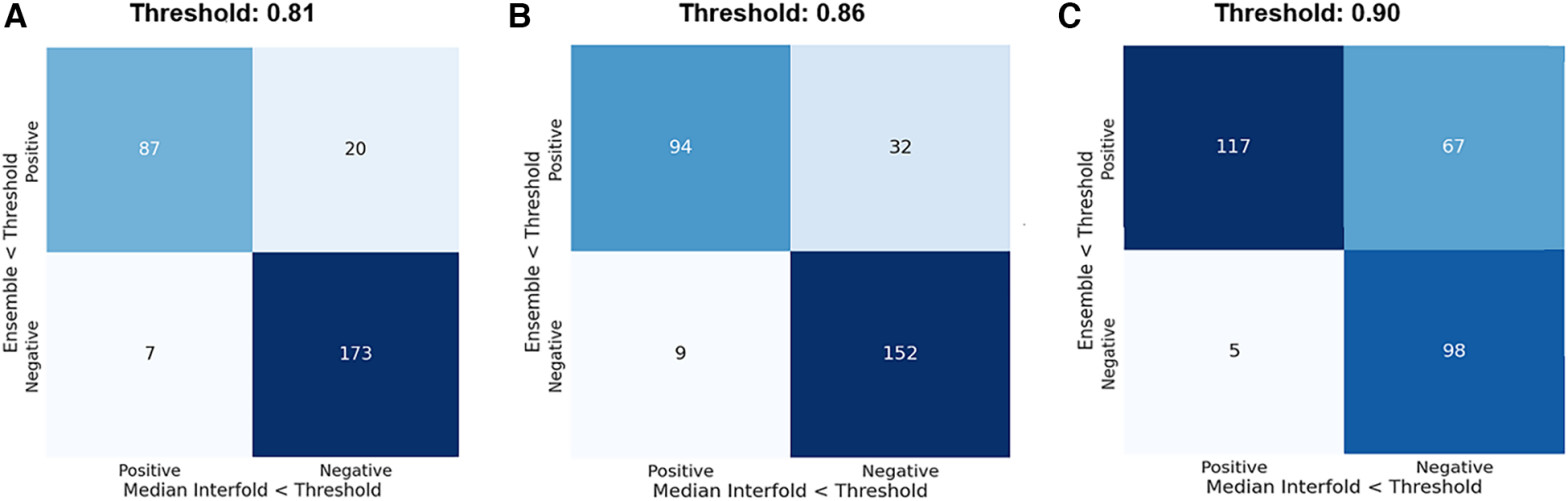
Confusion matrices for the three different median interfold dice score thresholds of (**A**) 0.9, (**B**) 0.86, and (**C**) 0.81. True positive (upper left) is defined as when flagged images (summary Interfold Dice < threshold) performed poorly (test ensemble < threshold). True negative (lower right) is defined as when non-flagged images (summary Interfold Dice > threshold) performed well (test ensemble > threshold). False positives (upper right) defined as when flagged images (summary Interfold Dice < threshold) performed well (test ensemble > threshold). False negatives (lower left) defined as when non-flagged images (summary Interfold Dice > threshold) performed poorly (test ensemble < threshold).

The mean test Dice score for tumor was 0.67 ± 0.36 with a significant improvement after removing the flagged cohort. As described above, we used a threshold of 0.90, where images with summarized Interfold Dices below this value were flagged. We also conducted a sensitivity analysis of two different arbitrary thresholds of 0.86 and 0.81, representing 90% and 95% of the original threshold. The full results of the impact of flagging with different summary metrics can be found in Table 5 and Figure 3. The confusion matrices of the three different thresholds can be found in Figure 4. As expected, a lower threshold will result in a smaller number of overall images being flagged and more false negatives, while a higher threshold will result in more images being flagged and more false positives. All the mean ensemble Dice values of the non-flagged cohorts were near the human interobserver value with small standard deviations values.

**Table 5:** Different summary metrics of interfold Dice flagged cohort—CT kidney tumor model on KiTS21 data.

Summary metrics	Number of flagged images	Flagged images mean ± standard deviation	Non-flagged images mean ± standard deviation
Mean	130	0.41 ± 0.38	0.89 ± 0.11
Median	122	0.37 ± 0.37	0.89 ± 0.10
Max	74	0.155 ± 0.251	0.85 ± 0.17
Min	162	0.49 ± 0.39	0.91 ± 0.07

**Figure 5:**
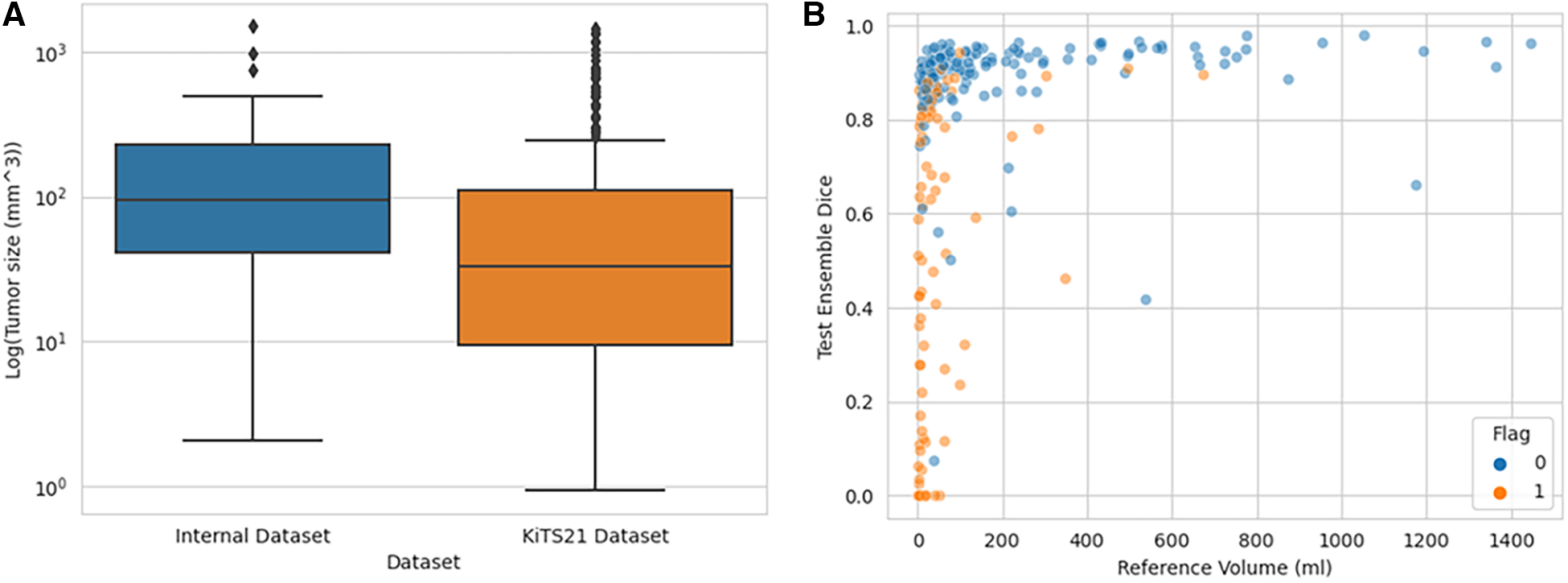
Distribution of tumor sizes in internally trained dataset vs. KiTS21, showing which KiTS21 data are flagged. (**A**) Boxplot graph demonstrating different tumor size distributions in CT datasets while (**B**) demonstrates how flagged images tended to be smaller tumor volumes.

As seen in Figure 5, the flagged images tended to be of tumors smaller than what was observed in the training set models:

### Qualitative assessment of flagged images

**Figure 6:**
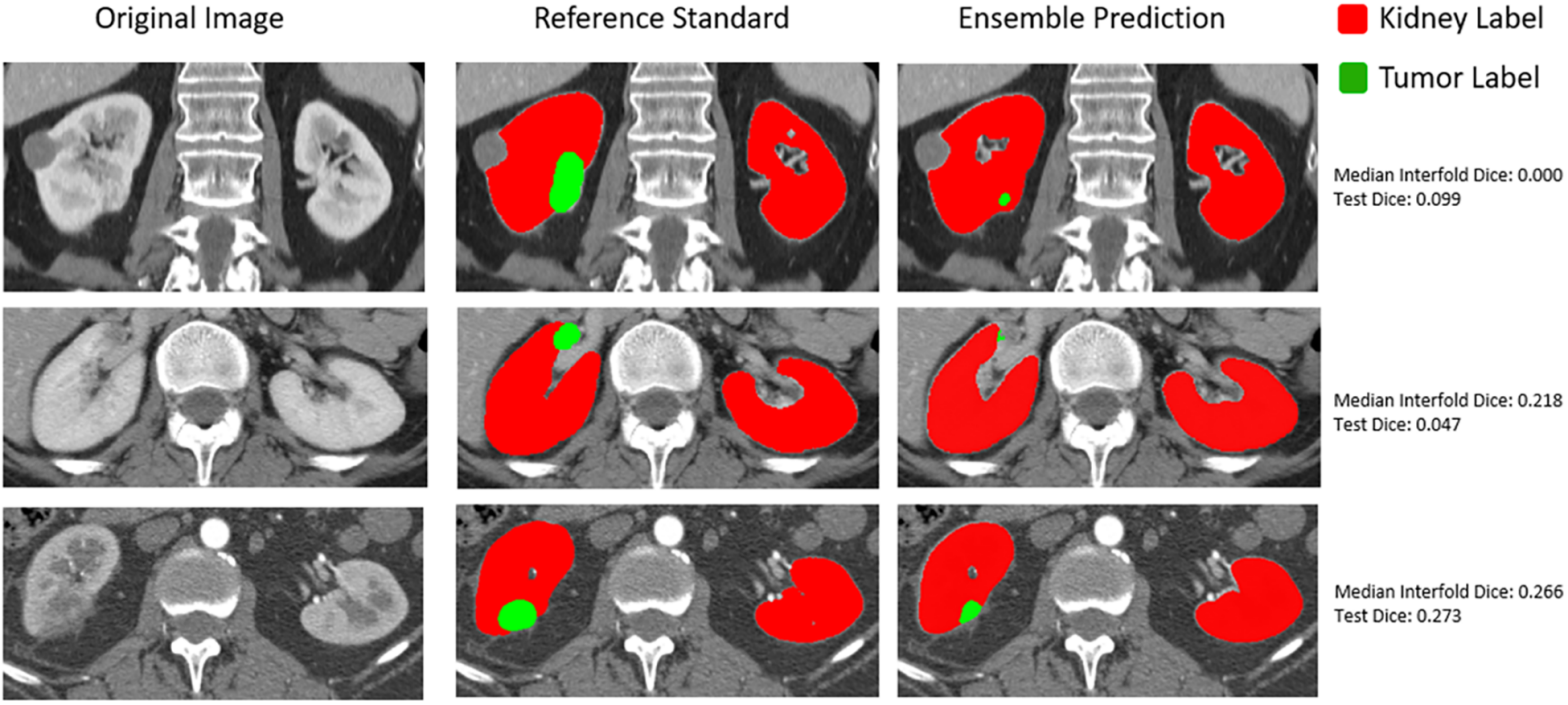
Qualitative assessment of outliers in internal CT tumor test set shown in the lowest left quadrant of Figure 4.

In addition to how out-of-distribution tumor size affected whether the model would have higher epistemic uncertainty and the impact on final ensemble test performance, we also qualitatively assessed flagged outliers. An important finding for the CT kidney and tumor internal data test set is that outliers tended to represent more difficult segmentation cases as opposed to corrupted images, which can be seen below in Figure 6.

## Discussion

The main goal of this paper was to leverage a state-of-the-art convolutional neural network framework to create a self-informed model that can be used to inform the user about the quality of the segmentation without comparing with any reference standard (i.e., applicable in scenarios where no reference standard exists). To identify poor-performing predictions, we compared sub-model predictions with each other and summarized them with different metrics to a single Interfold Dice score. This score was compared against published human interobserver thresholds to determine which images should be flagged in our hypothetical workflow. For segmentation tasks of tumors, flagged images tended to be the poorest-performing images, and the non-flagged predictions had significantly higher mean Dice values, showing less variability than the flagged predictions or the total predictions without flagging. Furthermore, we demonstrated by applying our internal model to the KiTS21 dataset that despite overall poor model performance, the non-flagged cohort still performed comparable with human interobserver values, while the images in the flagged cohort were generally of a smaller tumor size distribution than what was observed in the training dataset.

An intuitive understanding of why this method works relies on how cross-validation uses different distributions in training and validation folds to minimize overfitting on a single distribution. Despite seeing different distributions of data, we still expect predictions from different folds to resemble each other if the distribution of the test data is represented adequately so that examples are well distributed throughout the training and validation sets. However, in cases of out-of-distribution or near out-of-distribution, we expect greater prediction variance between folds, depending on the split of the limited relevant data in the training and validation sets. This prediction variance is a consequence of the folds not having adequate examples to converge to a ground truth prediction, resulting in a less sure prediction and, as demonstrated in the trials above, lower performance of the final ensemble model.

This application is a contribution to addressing the issue of epistemic uncertainty in the implementation of automated medical image segmentation models. Past quantitative work to detect out-of-distribution task data includes creating separate classification models to identify out-of-distribution data and quantifying uncertainty using Markov chain Monte Carlo methods (15, 16). Lakshminarayanan et al. (14) published a method most similar to the one presented here in comparing different ensemble models combined with adversarial training to identify out-of-distribution examples. Our study builds on this work by demonstrating a way to implement out-of-distribution detection in a medical image workflow using human interobserver values as thresholds for flagging. This real-time monitoring not only offers workflow implementers the ability to correct flagged examples, but it also alerts them to investigate and identify the causes of out-of-distribution data. In some cases, the out-of-distribution data may be due to corrupted input data or in fact represents a scenario of the need to update the model (e.g., data drift scenarios requiring continuous learning or other model update paradigms). Importantly, our method does not require the separate training or maintaining of separate upstream models, greatly simplifying its integration into clinical workflows.

A key limitation of this method is that it cannot correct for poor in-distribution training data. For example, the model may create a poor prediction with high certainty based on the training data that it sees. This problem is especially important to address in terms of entrenched biases that might be present in datasets (11, 28). Another limitation of our work is in deriving the thresholds used to evaluate whether the summarized Interfold Dices represent normal variability or lack of representative training data for the model to make a confident prediction. We used averaged published human interobserver values in this study to derive the thresholds. However, these values were derived from datasets with significant differences from the datasets that we were using. When implementing this method into a clinical pipeline, we advocate for researchers to conduct interobserver studies that are specific to their tasks and data to derive thresholds. Researchers may also consider investigating sensitivity analyses of different thresholds similar to what we have done in this study in order to balance the number of flagged images with the amount of false positive flagged images.

Regarding future directions, we plan to explore methods to determine ways to identify less obvious causes of higher epistemic uncertainty. In addition, we believe a prospective validation study demonstrating the method in real time is essential to assessing its utility for clinical implementation. Another direction that we are interested in is expressly stratifying flagged images by known concerning sources of bias, for example, ethnicity, to expressly investigate whether this bias may be present in our training data. Lastly, we have made our analysis code open-sourced and easily accessible for other investigators to determine its utility in different applications at the following link: https://github.com/TLKline/ai-in-the-loop.

## Conclusions

Comparing interfold sub-model predictions is an effective and efficient way to identify the epistemic uncertainty of a segmentation model, which is a key functionality for adopting these applications in clinical practice.

## Data Availability

The tabular data for our analysis with code can be found at https://colab.research.google.com/drive/1E4JBpl5X_9BXz_2AHUSM11z59CXD5DIc?usp=sharing.

## References

[1] Zhao C Han J Jia Y Gou F. Lung nodule detection via 3D U-Net and contextual convolutional neural network. 2018 International Conference on Networking and Network Applications (NaNA); 2018 Oct 12; Xi'an, China. New York, NY: IEEE (2018). p. 356–61.

[2] Hesamian MH Jia W He X Kennedy P. Deep learning techniques for medical image segmentation: achievements and challenges. J Digit Imaging. (2019) 32:582–96. 10.1007/s10278-019-00227-x 31144149 PMC6646484

[3] Yagi N Nii M Kobashi S. Abdominal organ area segmentation using u-net for cancer radiotherapy support. 2019 IEEE International Conference on Systems, Man and Cybernetics (SMC);2019 Oct 6; Bari, Italy. New York, NY: IEEE (2019). p. 1210–4.

[4] Siddique N Paheding S Elkin CP Devabhaktuni V. U-net and its variants for medical image segmentation: a review of theory and applications. IEEE Access. (2021) 9:82031–57. 10.1109/ACCESS.2021.3086020

[5] Rickman J Struyk G Simpson B Byun BC Papanikolopoulos N. The growing role for semantic segmentation in urology. Eur Urol Focus. (2021) 7:692–5. 10.1016/j.euf.2021.07.017 34417153

[6] Alakwaa W Nassef M Badr A. Lung cancer detection and classification with 3D convolutional neural network (3D-CNN). Int J Adv Comput Sci Appl. (2017) 8(8):2017.

[7] Heller N Isensee F Maier-Hein KH Hou X Xie C Li F The state of the art in kidney and kidney tumor segmentation in contrast-enhanced CT imaging: results of the KiTS19 challenge. Med Image Anal. (2021) 67:101821. 10.1016/j.media.2020.101821 33049579 PMC7734203

[8] Heller N Isensee F Trofimova D Tejpaul R Zhao Z Chen H The KiTS21 challenge: automatic segmentation of kidneys, renal tumors, and renal cysts in corticomedullary-phase CT. arXiv preprint. (2023) arXiv:2307.01984.

[9] Bilic P Christ P Li HB Vorontsov E Ben-Cohen A Kaissis G The liver tumor segmentation benchmark (LITS). Med Image Anal. (2023) 84:102680. 10.1016/j.media.2022.102680 36481607 PMC10631490

[10] Isensee F Jaeger PF Kohl SA Petersen J Maier-Hein KH. nnU-Net: a self-configuring method for deep learning-based biomedical image segmentation. Nat Methods. (2021) 18:203–11. 10.1038/s41592-020-01008-z 33288961

[11] Vayena E Blasimme A Cohen IGJPM. Machine learning in medicine: addressing ethical challenges. PLoS Med. (2018) 15:e1002689. 10.1371/journal.pmed.1002689 30399149 PMC6219763

[12] Shaw J Rudzicz F Jamieson T Goldfarb AJ. Artificial intelligence and the implementation challenge. J Med Internet Res. (2019) 21:e13659. 10.2196/13659 31293245 PMC6652121

[13] Swiler LP Paez TL Mayes RL. Epistemic uncertainty quantification tutorial. Proceedings of the 27th International Modal Analysis Conference; 2/9/2009 Sep 2; Orlando, FL. (2009).

[14] Lakshminarayanan B Pritzel A Blundell C. Simple and scalable predictive uncertainty estimation using deep ensembles. Adv Neural Inf Process Syst. (2017) 30. Available at: https://proceedings.neurips.cc/paper_files/paper/2017/file/9ef2ed4b7fd2c810847ffa5fa85bce38-Paper.pdf

[15] Ghoshal B Tucker A Sanghera B Lup Wong W. Estimating uncertainty in deep learning for reporting confidence to clinicians in medical image segmentation and diseases detection. Comput Intell. (2021) 37:701–34. 10.1111/coin.12411

[16] Abdar M Pourpanah F Hussain S Rezazadegan D Liu L Ghavamzadeh M A review of uncertainty quantification in deep learning: techniques, applications and challenges. Inf Fusion. (2021) 76:243–97. 10.1016/j.inffus.2021.05.008

[17] Zheng X Fu C Xie H Chen J Wang X Sham C-W. Uncertainty-aware deep co-training for semi-supervised medical image segmentation. Comput Biol Med. (2022) 149:106051. 10.1016/j.compbiomed.2022.106051 36055155

[18] Gottlich HC Gregory AV Sharma V Khanna A Moustafa AU Lohse CM Effect of dataset size and medical image modality on convolutional neural network model performance for automated segmentation: a CT and MR renal tumor imaging study. J Digit Imaging. (2023) 36:1–12. 10.1007/s10278-023-00804-1 36932251 PMC10406754

[19] Kline TL Korfiatis P Edwards ME Blais JD Czerwiec FS Harris PC Performance of an artificial multi-observer deep neural network for fully automated segmentation of polycystic kidneys. J Digit Imaging. (2017) 30:442–8. 10.1007/s10278-017-9978-1 28550374 PMC5537093

[20] Van Gastel MD Edwards ME Torres VE Erickson BJ Gansevoort RT Kline TL. Automatic measurement of kidney and liver volumes from MR images of patients affected by autosomal dominant polycystic kidney disease. J Am Soc Nephrol. (2019) 30:1514–22. 10.1681/ASN.2018090902 31270136 PMC6683702

[21] Denic A Elsherbiny H Mullan AF Leibovich BC Thompson RH Archila LR Larger nephron size and nephrosclerosis predict progressive CKD and mortality after radical nephrectomy for tumor and independent of kidney function. J Am Soc Nephrol. (2020) 31:2642. 10.1681/ASN.2020040449 32938650 PMC7608955

[22] Yushkevich PA Piven J Hazlett HC Smith RG Ho S Gee JC User-guided 3D active contour segmentation of anatomical structures: significantly improved efficiency and reliability. Neuroimage. (2006) 31:1116–28. 10.1016/j.neuroimage.2006.01.015 16545965

[23] Beck A Goetsch L Dumontet C Corvaïa N. Strategies and challenges for the next generation of antibody–drug conjugates. Nat Rev Drug Discov. (2017) 16:315–37. 10.1038/nrd.2016.268 28303026

[24] Taha AA Hanbury A. Metrics for evaluating 3D medical image segmentation: analysis, selection, and tool. BMC Med Imaging. (2015) 15:1–28. 10.1186/s12880-015-0042-7 26263899 PMC4533825

[25] Müller S Farag I Weickert J Braun Y Lollert A Dobberstein J Benchmarking Wilms’ tumor in multisequence MRI data: why does current clinical practice fail? Which popular segmentation algorithms perform well? J Med Imaging. (2019) 6:034001. 10.1117/1.JMI.6.3.034001 PMC6639723 31338388

[26] Nguyen K Schieda N James N Mcinnes MD Wu M Thornhill RE. Effect of phase of enhancement on texture analysis in renal masses evaluated with non-contrast-enhanced, corticomedullary, and nephrographic phase–enhanced CT images. Eur Radiol. (2021) 31:1676–86. 10.1007/s00330-020-07233-6 32914197

[27] Haarburger C Müller-Franzes G Weninger L Kuhl C Truhn D Merhof D. Radiomics feature reproducibility under inter-rater variability in segmentations of CT images. Sci Rep. (2020) 10:1–10. 10.1038/s41598-020-69534-6 32728098 PMC7391354

[28] Braveman P. Health disparities and health equity: concepts and measurement. Annu Rev Public Health. (2006) 27:167–94. 10.1146/annurev.publhealth.27.021405.102103 16533114

